# Hypomethylation of Serum Blood Clot DNA, but Not Plasma EDTA-Blood Cell Pellet DNA, from Vitamin B12-Deficient Subjects

**DOI:** 10.1371/journal.pone.0065241

**Published:** 2013-06-13

**Authors:** Eoin P. Quinlivan, Krista S. Crider, Jiang-Hui Zhu, David R. Maneval, Ling Hao, Zhu Li, Sonja A. Rasmussen, R. J. Berry, Lynn B. Bailey

**Affiliations:** 1 Biomedical Mass Spectrometry Laboratory, Clinical and Translational Science Institute, University of Florida, Gainesville, Florida, United States of America; 2 National Center on Birth Defects and Developmental Disabilities, Centers for Disease Control and Prevention, Atlanta, Georgia, United States of America; 3 Department of Surveillance Program and Risk Assessment, China National Center for Food Safety Risk Assessment, Beijing, China; 4 Food Science and Human Nutrition Department, University of Florida, Gainesville, Florida, United States of America; 5 Centers for Disease Control and Prevention–Global Aids Program, China Office, Beijing, China; 6 Peking University Health Science Center, Beijing, China; 7 Department of Foods and Nutrition, University of Georgia, Athens, Georgia, United States of America; Chinese Academy of Science, China

## Abstract

Vitamin B12, a co-factor in methyl-group transfer, is important in maintaining DNA (deoxycytidine) methylation. Using two independent assays we examined the effect of vitamin B12-deficiency (plasma vitamin B12<148 pmol/L) on DNA methylation in women of childbearing age. Coagulated blood clot DNA from vitamin B12-deficient women had significantly (p<0.001) lower percentage deoxycytidine methylation (3.23±0.66%; n = 248) and greater [3 H]methyl-acceptance (42,859±9,699 cpm; n = 17) than DNA from B12-replete women (4.44±0.18%; n = 128 and 26,049±2,814 cpm; n = 11) [correlation between assays: r = –0.8538; p<0.001; n = 28]. In contrast, uncoagulated EDTA-blood cell pellet DNA from vitamin B12-deficient and B12-replete women exhibited similar percentage methylation (4.45±0.15%; n = 77 vs. 4.47±0.15%; n = 47) and [3 H]methyl-acceptance (27,378±4,094 cpm; n = 17 vs. 26,610±2,292 cpm; n = 11). Therefore, in simultaneously collected paired blood samples, vitamin B12-deficiency was associated with decreased DNA methylation only in coagulated samples. These findings highlight the importance of sample collection methods in epigenetic studies, and the potential impact biological processes can have on DNA methylation during collection.

## Introduction

Deoxycytidine methylation (dC → 5 mdC) patterns are transmitted through the germ line [1] and occur primarily at the 5′-dC of the 5′-deoxycytidine-deoxyguanosine-3′ (CpG) motif in somatic cells [2], [3], [4]. Methylation of transcriptional start sites can regulate gene expression [5] by directly inhibiting transcription factor binding [6] and by initiating chromatin recruitment [7]. This suppression may play a critical role in regulating autosomal gene inheritance [8], X-chromosome inactivation [9], [10], and inactivation of repetitive elements [10], [11], [12] such as long interspersed nuclear elements (LINEs), endogenous retroviruses, and satellite sequences which comprise ∼35% of the genome [13]. Conversely, methylation of the gene body [14], [15], [16] and subsequent chromatin recruitment may promote transcription [17]. Centromere methylation may increase chromosomal stability [18], [19], while the greater methyl-density [3], [20] and nucleosome-occupancy [21] found in exons, and the sharp transition in methyl-density at the intron-exon boundary [3], [20], may act as a marker for splicing [22].

**Figure 1:**
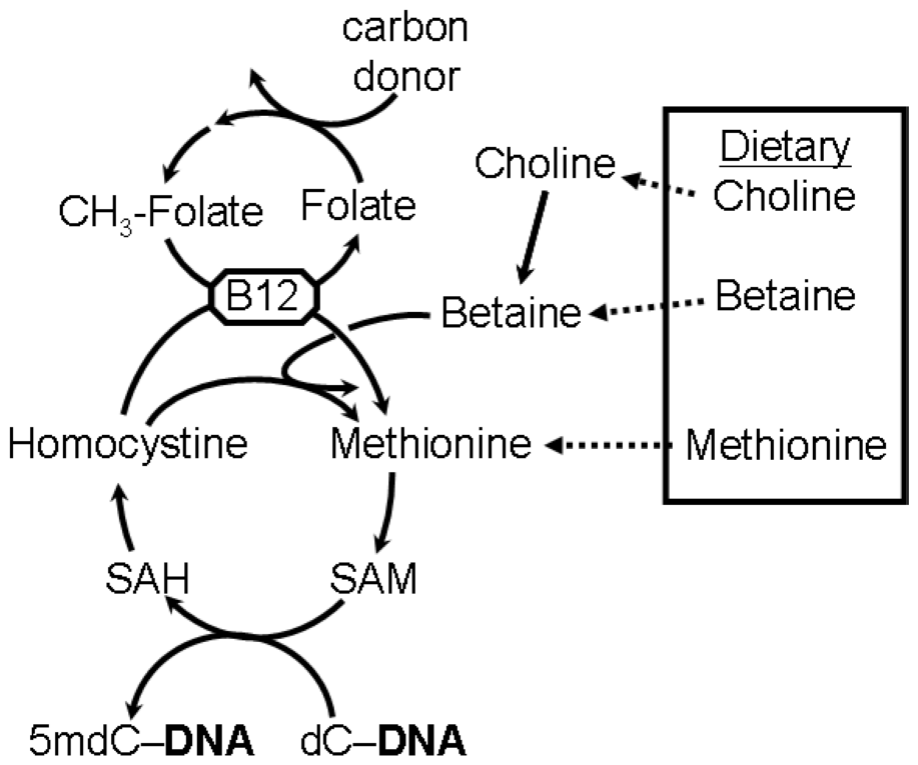
Schematic of the One-Carbon cycle. Vitamin B12 is a co-factor in the transfer of methyl-groups (CH_3_−) from folate to methionine for use *in situ* methylation of deoxycytidine (dC) to in 5-methyldeoxycytidine (5 mdC) in DNA. Choline, betaine and methionine can be derived from the diet or synthesized *in vivo*. SAM: S-adenosylmethionine; SAH: S-adenosylhomocysteine.

Differences in epigenetic patterns between individuals with similar genetic backgrounds may account for differences in health outcomes [23], [24], [25] and, as a consequence, can contribute to the etiology of numerous health-related conditions including infertility, stroke, atherosclerosis, obesity, insulin resistance, kidney disease, cancer, neural tube defects and autoimmunity [26], [27], [28], [29], [30], [31]. Meanwhile, nutritional alteration of the epigenome *in utero* may be persistent [32], [33], [34] and may contribute to the health [34], [35], [36], [37] of the offspring in later life. The methyl-groups used for DNA methylation [38], [39] are derived from preformed dietary sources (choline, betaine, methionine) or are generated *de novo* via the folate cycle (Fig. 1) – with vitamin B12 (cobalamin) acting as co-factor in the transfer of methyl-groups from the folate cycle to the methylation cycle. Data from a number of small-scale human studies [40], [41], [42] suggest that folate depletion and/or repletion can affect DNA methylation in peripheral blood cells. Likewise, Friso *et al.* [43] reported that methylenetetrahydrofolate reductase (MTHFR) 677 C→T homozygous subjects with a sub-optimum folate status had significantly less 5-methyldeoxycytidine (5 mdC) than wild-genotype subjects (regardless of folate status) or folate-replete homozygous subjects. Our research group previously reported differences in DNA methylation level between DNA isolated from coagulated blood clots (which exhibited decreased 5 mdC) vs. uncoagulated EDTA-blood cell pellets (which exhibited no change in 5 mdC) that were dependent on folic acid supplementation and the discontinuation of supplementation in a population-based trial of folic acid supplementation [44].

In humans, prolonged vitamin B12-deficiency can result in numerous clinical ramifications including nerve damage, anemia, digestive and cognitive problems [45]. Additionally, vitamin B12-deficiency might be a risk factor for neural tube defects [46], [47]. Despite the important role vitamin B12 plays in one-carbon metabolism (Fig. 1) and DNA methylation, relatively little has been reported on the effect of vitamin B12-deficiency on DNA methylation. Rodents fed vitamin B12-deficient diets [48], [49], [50] or a diet deficient in several B-vitamins [51] exhibited decreased DNA methylation. In a single case study, Smulders *et al.* [52] reported that DNA methylation increased 22% in a vitamin B12-deficient human after vitamin B12 intervention.

The primary objective of the present study was to describe the effect of vitamin B12-deficiency on global DNA methylation in a female Northern Chinese population of reproductive age. This population has a high (∼21%) prevalence of vitamin B12-deficiency (<148 pmol/L) [53], [54].

## Methods

### Ethics Statement

Participants provided oral informed consent. This was permissible as the study posed no more than minimal risk of harm to the participant and was documented with a signature of the consenting investigator as was culturally appropriate for the research setting and involved no procedure for which written consent was normally required outside of a research setting. The study and consent procedures including a waiver for the documentation of informed consent as set forth in 45CFR46.117(c) were approved by the Centers for Disease Control and Prevention Institutional Review Board and the Ethical Committee on Biomedical Research Involving Human Subjects of the Health Science Center, Peking University. The samples used in this study were screening samples from a folic acid intervention study [44], [55], [56] - the screening/baseline samples were used to identify suitable subjects who met the inclusion criteria to participate in the intervention study (the intervention trial is registered at clinicaltrials.gov: NCT00207558). No intervention was performed on the subjects prior to collection of the samples used for this manuscript.

All of the vitamin B12-deficient (plasma vitamin B12<148 pmol/L) subjects, identified from the screening samples, were excluded from participation in the subsequent intervention trial and were referred for treatment.

### Setting and Inclusion Criteria for Participants

Women were recruited from six townships of Xianghe County, Hebei Province, in Northern China, for a population-based folic acid intervention study [44], [55], [56]. Participants were not exposed to dietary sources of folic acid since folic acid-fortified foods were not available in China. Eligibility requirements included: residence in the townships; not pregnant or breastfeeding, and not plan to become pregnant in the next 9 months; use an IUD for contraception; have a child 2–4 y of age; have no chronic diseases; no supplement use within the last 3 months, and no current prescription medication use [55].

### Blood Collection Procedures

Fasting blood samples from each participant were collected into 7 ml tubes with EDTA, and 3 ml tubes containing no anticoagulants (Vacutainer; Becton Dickinson).

Coagulated blood clots (blood clots) were prepared by allowing the blood tubes, containing no anticoagulant, to stand at room temperature for 1–2 hours, as previously described [44]. Sera were separated by centrifugation at 2000 × g for 15 minutes at 4°C. After the sera were removed the blood clots were frozen and stored at −20°C.

Uncoagulated blood cell pellets (EDTA-blood cell pellets) were prepared within 1 hour of collection by centrifuging the EDTA blood tubes at 2,000 × g for 15 minutes at 4°C. After the plasma was removed the uncoagulated EDTA-blood cell pellet was frozen at −20°C.

Blood clots and EDTA-blood cell pellets were stored at −20°C before being transported on dry ice to the central laboratory of Peking University Health Science Center where they were stored at −70°C.

### Biochemical Characteristics

Plasma vitamin B12 was measured in duplicate samples by using the Quantaphase II radioassay (Bio-Rad Laboratories; Product # 191–1044). Subjects were characterized as vitamin B12-deficient (plasma vitamin B12<148 pmol/L; n = 305) or vitamin B12-replete (plasma vitamin B12>148 pmol/L; n = 1139).

Plasma and RBC folate concentrations were measured by microbiological assay [57] while plasma homocysteine was assayed by HPLC with fluorometric detection [58].

### Sampling for % 5 mdC Analysis

Eligible subjects (n = 1,702) were enrolled in the study and provided blood samples. Subjects with B12-deficiency (<148 pmol/L) and clot DNA available were included as B12-deficient (n = 248). Subjects with normal B12 status (plasma B12>148 pmol/L) and normal hemoglobin concentration (>120 g/L) were included as non-deficient controls (n = 128) and were selected to have an even distribution of *MTHFR* genotypes as previously reported [44]. *MTHFR TT* genotype was not associated with global DNA methylation at enrollment in either the control [44] or B12-deficient participants (not shown) in clots and therefore was not controlled for statistically in these analyses.

### Genomic DNA Extraction

Blood clots were shipped to the University of Florida on dry ice for genotyping and for percentage deoxycytidine methylation determination. After the initial blood clot % 5 mdC analysis was completed, a subset of the EDTA-blood cell pellets, which had been retained at the Peking University Health Science Center, was shipped on dry ice to the University of Florida for % 5 mdC analysis.

Genomic DNA was extracted from stored frozen blood clots or EDTA-blood cell pellets according to the manufacturer’s instructions using a commercially available blood DNA purification kit (Gentra Puregene, Qiagen, Valencia, CA). Samples were hydrated in TE buffer, and DNA concentration determined by fluorescent dye binding assay (Quant-iT, Invitrogen; Carlsbad, CA) and adjusted to 20 ng/µl. No systematic attempt was made to determine or compare DNA yield from each blood sample matrix. However, the amount of DNA extracted was within the expected range (based on the extraction kit handbook and experience extracting similar samples) and did not appear to differ between the two matrices, nor between vitamin B12-deficient or replete samples.

### Percentage Deoxycytidine Methylation

After digesting the DNA to nucleosides [59] the concentrations of dC and 5 mdC were determined by LC-MS/MS using biosynthetic [^15^N_3_]dC and [^15^N_3_]5 mdC internal standards [60]. Percentage deoxycytidine methylation (% 5 mdC) was calculated as % 5 mdC = [5 mdC]/([dC]+[5 mdC])×100. Inter- and intra-assay variation (relative standard deviation; n = 6) for the assay was <2.5%.

### Enzymatic [^3^H]Methyl-acceptance

The extent to which the CpG motif in DNA was unmethylated was determined by [^3^H]methyl-acceptance assay using a modification of the method of Balaghi and Wagner [61], as previously described [40].

Due to the large inter-assay variability reported [40] for the [^3^H]methyl-acceptance assay the number of DNA samples analyzed was limited to the number that could be assayed within a single assay. Consequently, 17 pairs of DNA from vitamin B12-deficient subjects and 11 pairs from vitamin B12-replete subjects were analyzed within the same assay. The vitamin B12-deficient paired samples were chosen (based on the %MdCyt results of their blood clot DNA) so as to give a range of DNA methylation levels.

### Assay Comparison

Regression analysis was performed to determine the relationship between the [^3^H]methyl-acceptance and % 5 mdC blood clot DNA results. Data from the vitamin B12-deficient (n = 17) and replete (n = 11) groups were included in the regression analysis.

### Statistical Analysis

Arithmetic means and standard deviations were calculated to compare participant characteristics. Paired t-tests were used to test for matrix effects within the vitamin B12-deficient or replete groups; pooled t-tests were used to test for differences between vitamin B12-deficient and replete groups. For all tests that did not reach statistical significance, power analyses [62] were conducted to estimate minimum detectable percentage difference in means (power >80%). All variables were log transformed to achieve normal distributions for regression analysis. Univariate and multivariate linear regression was conducted with SPSS (SPSS Inc. Released 2009. PASW Statistics for Windows, Version 18.0, Chicago: SPSS Inc.).

## Results

### Biochemical Characteristics

Physical and biochemical characteristics of the subjects included in this analysis are presented in (Table 1).

**Table 1:** Basic Biochemical Characteristics of Subjects.

	B12-replete^1^	B12-deficient^2^	
	mean (SD)	mean (SD)	Pooled t-test
n	128	248	
Age (y)	30.3 (4.0)	31.5 (4.1)	0.011
Body mass index (kg/m^2^)	24.0 (4.4)	24.1 (3.8)	0.704
Plasma vitamin B12 (pmol/L)	284 (106)	113 (28)	<0.001
Plasma folate (nmol/L)	10.1 (4.9)	9.6 (5.7)	0.399
Red cell folate (nmol/L)	683 (315)	535 (224)	<0.001
Total plasma homocysteine (µmol/L)	8.7 (5.6)	13.3 (11.0)	<0.001

1 Subjects with plasma vitamin B12 more than 148 pmol/L.

2 Subjects with plasma vitamin B12 less than 148 pmol/L.

### Percentage Deoxycytidine Methylation

Blood clot DNA exhibited a significantly lower (p<0.001) percentage deoxycytidine methylation (% 5 mdC) in vitamin B12-deficient samples compared to vitamin B12-replete samples (Table 2), such that there was little overlap in % 5 mdC between the vitamin B12-deficient and replete samples (Fig. S1). Reanalysis of different subsets of blood clot DNA from vitamin B12-deficient subjects over several months demonstrated (Fig. S2) the reproducibility of the % 5 mdC assay (r >0.9; p<0.001).

**Table 2:** Effect of vitamin B12-deficiency on % 5 mdC^1^ for DNA extracted from coagulated blood clots^2^ and uncoagulated EDTA-blood cell pellets^3^.

	All Samples^4^		Matched Samples^5^			
		Blood clot		Blood pellet	Blood clot	
	n	mean (SD)	n	mean (SD)	mean (SD)	Pooled t-test (p)
B12-replete^6^	128	4.44 (0.18)	47	4.47 (0.15)	4.45 (0.17)	0.451*
B12-deficient^7^	248	3.23 (0.66)	77	4.45 (0.15)	2.88 (0.86)	<0.001
Paired t-test		<0.001		0.367*	<0.001	

1 Methyldeoxycytidine as a percentage of total deoxycytidine in DNA digests, measured by LC-MS/MS (see Methods).

2 Post-centrifugation, blood clots were retained after serum was removed from serum blood tubes.

3 Post-centrifugation, blood cell pellets were retained after plasma was removed from EDTA-blood tubes.

4 Results for all assayed blood clot DNA.

5 Matched data for blood clot and EDTA-blood cell pellet DNA from the same subjects.

6 Subjects with plasma vitamin B12 more than 148 pmol/L.

7 Subjects with plasma vitamin B12 less than 148 pmol/L.

* Power analysis: >80% confidence to detect a 2% difference in means [62].

Analysis of DNA extracted from EDTA-blood cell pellets detected no differences in % 5 mdC (Table 2) between the vitamin B12-deficient and replete subjects.

### Enzymatic [^3^H]Methyl-acceptance Assays

Blood clot DNA from vitamin B12-deficient subjects exhibited significantly (p<0.001) greater [^3^H]methyl-acceptance than blood clot DNA from vitamin B12-replete subjects (Table 3). In contrast, there was no apparent difference in [^3^H]methyl-acceptance between the vitamin B12-deficient EDTA-blood cell pellet DNA and either the vitamin B12-replete blood clot or EDTA-blood cell pellet DNA.

**Table 3:** Effect of vitamin B12-deficiency on % 5 mdC^1^ and [3 H]methyl-acceptance capacity^2^ for DNA extracted from coagulated blood clots^3^ and uncoagulated EDTA-blood cell pellets^4^.

		% 5 mdC			[3 H]methyl-acceptance (cpm/250 ng)		
		[mean (SD)]			[mean (SD)]		
	n	Clot DNA	Pellet DNA	Paired t-test	Clot DNA	Pellet DNA	Paired t-test
B12-replete^5^	11	4.41 (0.12)	4.44 (0.12)	0.311*	26,049 (2,814)	26,610 (2,292)	0.626**
B12-deficient^6^	17	2.63 (1.16)	4.33 (0.16)	<0.001	42,859 (9,699)	27,378 (4,094)	<0.001
Pooled t-test		<0.001	0.070*		<0.001	0.577***	

1 Methyldeoxycytidine as a percentage of total deoxycytidine in DNA digests, measured by LC-MS/MS (see Methods).

2 Capacity of DNA to incorporate [3 H] from [3 H-methyl]SAM (cpm incorporated per 250 ng DNA) in the presence of Sssi DNA methyltransferase (see Methods).

3 Post-centrifugation, blood clots were retained after serum was removed from plain blood tubes.

4 Post-centrifugation, blood pellets were retained after plasma was removed from EDTA blood tubes.

5 Subjects with plasma vitamin B12>148 pmol/L.

6 Subjects with plasma vitamin B12<148 pmol/L.

Power analysis: >80% power to detect a *4%, **10%, or ***15% difference in means [62].

### Assay Comparison

**Figure 2:**
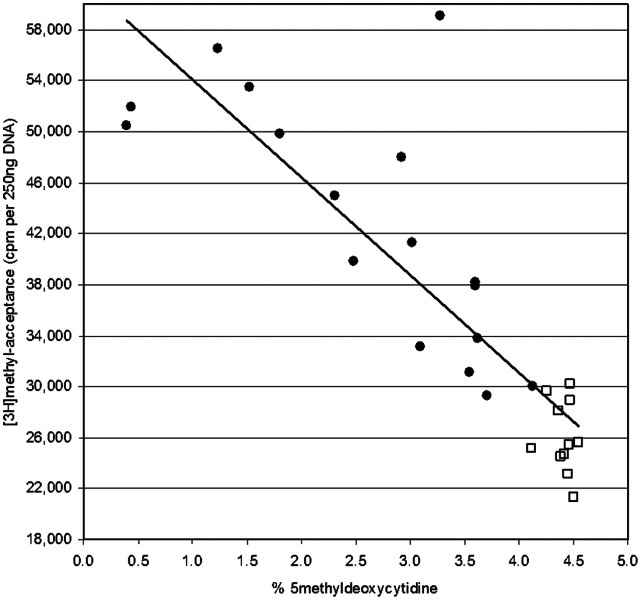
Plot of % 5-methyldeoxycytidine versus [^3^H]methyl-acceptance capacity of DNA extracted from coagulated blood clots. DNA samples were from (•) vitamin B12-deficient (plasma vitamin B12<148 pmol/L) or (□) vitamin B12-replete subjects (plasma vitamin B12>148 pmol/L). % 5-methyldeoxycytidine: 5-methyldeoxycytidine as a percentage of total deoxycytidine in DNA digests as measured by LC-MS/MS (see Methods). [3 H]Methyl-acceptance: [3 H] (cpm) from [3 H-methyl]SAM incorporated into 250 ng of DNA in the presence of Sssi DNA methyltransferase.

When % 5 mdC and [^3^H]methyl-acceptance results, for blood clot DNA (17 vitamin B12-deficient and 11 vitamin B12-replete samples), were plotted against each other (Fig. 2) a significant inverse correlation (r = –0.8538; p<0.001; n = 28) was observed.

### Linear Regression

To further explore the decrease in % DNA methylation observed in the B12-deficient participants’ coagulated samples, univariate and multivariate linear regression was used (n = 248). Factors examined included: plasma vitamin B12 concentration (within deficiency), RBC folate concentrations, hemoglobin, age, body mass index (BMI) and homocysteine concentration. In multivariate linear regression models, DNA methylation level was only statistically significantly associated with homocysteine (p = 0.04; β = −0.145) and hemoglobin concentrations (p = 0.002; β = −0.210).

## Discussion

We observed a significantly different response to vitamin B12-deficiency in the two simultaneously collected blood sample matrices (blood clot vs. EDTA-blood cell pellet) studied; namely, blood clot DNA exhibited hypomethylation, while EDTA-blood cell pellet DNA did not. The extent (Table 2) and range (Fig. 2, Fig. S1) of DNA hypomethylation observed were of a similar magnitude to that previously reported [43] in elderly humans, and were comparable to those observed in rodents fed vitamin B12-deficient [48], [49], [50] or multivitamin-deficient [51] diets. The differences in DNA methylation between the two sample matrices were confirmed using two independent measures of DNA methylation: a quantitative determination of the extent dC methylation (the % 5 mdC assay) and a qualitative determination of non-methylated CpG (the [3 H]methyl-acceptance assay).

We reported a similar effect of sample matrix on % 5 mdC in the healthy (non-anemic, non-B12-deficient) participants following folic acid supplementation and discontinuation of supplementation [44]; DNA extracted from blood clots exhibited significant changes in % 5 mdC, while EDTA-blood cell pellet DNA, collected at the same time from the same subjects, exhibited no change in % 5 mdC. In the present study, we were able to extensively validate (Table 3; Fig. 2) these % 5 mdC results by comparing the results with those obtained using an independent assay ([3 H]methyl-acceptance assay). Additionally, we found that this phenomenon occurs in response to another perturbation to one-carbon metabolism – vitamin B12-deficiency.

This disparity in response between the two blood sample matrices is of concern as it suggests that sample collection may have as significant an impact on apparent DNA methylation as *in vivo* biochemical conditions. While we examined only two sample matrices, coagulated blood clots and uncoagulated EDTA-blood cell pellets, questions must be asked whether DNA methylation is as labile in other blood matrices (e.g., whole blood, blood spots), using other anticoagulants (e.g., citrate, heparin), in other tissue types (e.g., cell biopsies, cell culture, buccal cells) or using different sample preparation techniques. This effect should be taken into consideration when comparing epigenetic markers that were determined using different methods.

The difference in % 5 mdC between sample matrices, cannot be explained by a difference in DNA quality (e.g., denatured, fragmented), as the DNA is digested to nucleosides as part of the assay, and so is independent of DNA quality. Poor DNA quality can decrease [3 H]methyl-acceptance (as the enzyme only methylates double stranded DNA). If the EDTA-blood clot DNA was hypomethylated, a decrease in DNA quality might explain why the [3 H]methyl-acceptance was not elevated. However, the magnitude of the decrease in DNA quality would have to be just enough so that the [3 H]methyl-acceptance appeared normal (Table 3), and would not explain the % 5 mdC result.

The extensive use of the [3 H]methyl-acceptance assay in this study, and the inverse correlation (r = –0.8538; p<0.001) observed between it and the % 5 mdC assay (Fig. 2) provides important information about the samples. Firstly, it independently confirms the results of the % 5 mdC assay: that blood clot DNA from vitamin B12-deficient subjects appears hypomethylated, while EDTA-blood cell pellet DNA from the same subjects does not. Secondly, it suggests that the difference in methylation between the sample matrices is primarily due to interconversion between dC and 5 mdC; rather than the simple loss or gain of 5 mdC, or the modification of 5 mdC (e.g., to 5-hydroxymethyl-dC (5 hmdC) [63], [64]). Whether dC↔5 mdC interconversion occurred *in vivo* or during sample collection and processing is not clear.

### Possible Mechanisms for the Differences in Methylation Observed between Sample Matrices

#### Difference in blood cell composition between blood sample matrixes

The DNA methylation is similar in mononuclear and polynuclear peripheral blood cells [65], and in the white blood cell population as a whole. Therefore, differences in nucleated cell populations between the blood clot and EDTA-blood cell pellet are unlikely to explain the differences in DNA methylation.

#### DNA methylation became altered in the EDTA-blood cell pellet

Use of EDTA as anticoagulant may have inadvertently triggered DNA ‘remethylation’: By scavenging metal cations EDTA may decreased inhibition of the DNA methyltransferases [66]. Chelation of magnesium by EDTA may also decrease interaction between DNMT3L and the DNA methyltransferases [67], changing the mode of DNA methylation from processive to distributive [68], [69].

However, it is not clear where the methyl-groups needed to ‘remethylate’ the DNA would come from: The human genome is comprised of an estimated 6.16×10^9^ bases [70] of which ∼1.25×10^9^ (∼2.1×10^−15^ moles) are dC [71]. To methylate 1% of the dC to 5mdC (e.g., to elevate the mean % 5 mdC from 3.4% to 4.4%) would require 2.1×10^−17^ moles of SAM per cell. Ignoring all the other methyltransferase reaction in leukocytes [72], this is 4.5 times greater than the SAM concentration in lymphocyte (4.7×10^−18^ moles/cells*) [73] and exceeds the SAM synthesis rate in mononuclear blood cells (∼2×10^−17^ mol/hr/cell) [74], [75], [76]. This imbalance between SAM usage and availability may be particularly acute in vitamin B12-deficiency as the activity of both methionine synthase [77], [78], [79], [80] and SAM synthase [78] may be decreased, thereby decreasing SAM concentrations [78], [80].

[*NOTE: there is a typographical error in [Table 3] of Reference [73] – the SAM concentration should read ‘pmol/10^6^’ cells, not ‘nmol/10^6^’ (S.J. James, personal communication).].

#### DNA methylation became altered in the blood clot

**Figure 3:**
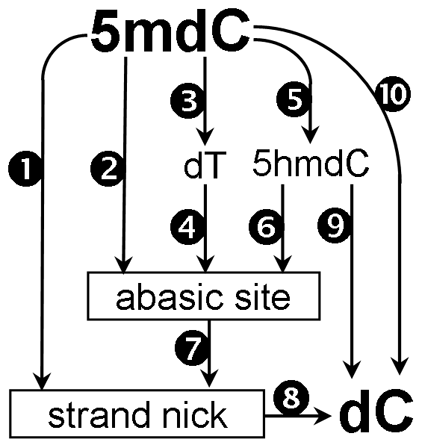
Putative mechanisms for the conversion of 5 mdC to dC in DNA. Reactions: 1) 5 mdC nucleases [91]; 2) 5 mdC glycosylase [92], [93], [94]; 3) 5 mdC deaminase [84], [87], [95], [96]; 4) thymine-DNA glycosylases [97]; 5) 5 mdC hydroxylase [63], [64]; 6) 5 hmC glycosylase [98]; 7) AP endonuclease/phosphodiesterase [99], [100]; 8) DNA polymerase/DNA ligase [100]; 9) 5 hmdC dehydroxymethylase [88], [89]; 10) DNA demethylase [101], [102], [103].

Several pathways [Fig 3] have been proposed to explain the active conversion of 5 mdC to dC observed in embryogenesis [81], [82], in some cancers [83], and during reactivation of epigenetically suppressed or imprinted genes [84], [85], [86]. Some of these reactions (5 mdC deaminase (Fig. 3; Reaction 3) [84], [87] and 5 hmdC dehydroxymethylase (Fig. 3; Reaction 5) [88], [89] only occur at low SAM concentrations suggesting that DNA demethylation may have occur only in vitamin B12-deficient clots. Alternatively, DNA demethylation and remethylation may be ubiquitous in all blood clots, but the methylation capacity of vitamin B12-deficient cells may have been insufficient [90] to restore normal DNA methylation levels.

### Conclusions

These findings raise questions concerning whether DNA methylation can be influenced by the sample collection method. In addition to the two blood matrices described, these findings might have implications for other tissue types. If vitamin B12-deficiency causes DNA hypomethylation *in vivo* this level of hypomethylation would have clear epigenetic implications. Moreover, this study suggests that the hypomethylation observed in blood clot DNA (whether it is representative of *in vivo* methylation or not) might be a good biomarker for perturbations to the methylation cycle and has possible implications for the health impacts of alterations in one-carbon metabolism.

## Supporting Information

Figure S1 **Percentage 5-methyldeoxycytidine in blood clot DNA.** DNA from (◊) vitamin B12-replete (plasma vitamin B12>148 pmol/L) and (♦) vitamin B12-deficient (plasma vitamin B12<148 pmol/L) subjects. Hashmarks to the outside of each group represent mean ± SD. Spacing along the X-axis is used only to show individual data points. See Text for further detail.
(PDF)

Figure S2 **Reproducibility of the 5-methyldeoxycytidine LC-MS/MS assay.** Subsets of blood clot DNA from vitamin B12-deficient subjects (plasma vitamin B12<148 pmol/L) were analyzed at different times over a two-year period and compared to results from the primary (n = 248) DNA assay. % 5-methyldeoxycytidine: 5-methyldeoxycytidine as a percentage of total deoxycytidine in DNA digests as measured by LC-MS/MS (see Methods). The broken line represents unity (X = Y). See Text for further details.
(PDF)
