# Supplementary figures and images for: Hypomethylation of Serum Blood Clot DNA, but Not Plasma EDTA-Blood Cell Pellet DNA, from Vitamin B12-Deficient Subjects
Source: PLoS One. 2013 Jun 13;8(6):e65241. doi: 10.1371/journal.pone.0065241 (PMC3681792; doi:10.1371/journal.pone.0065241)

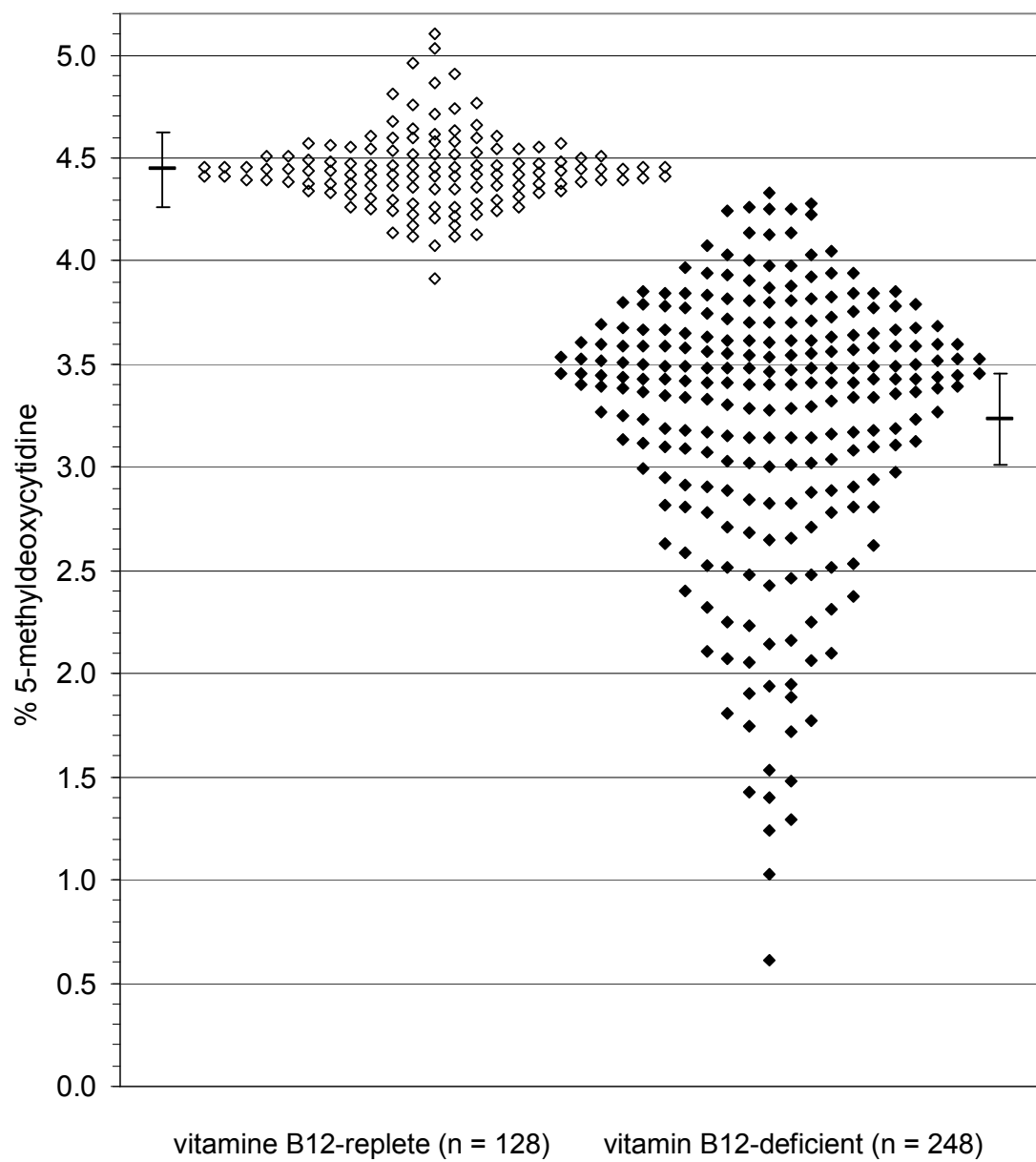

Supplement: Figure S1 — Percentage 5-methyldeoxycytidine in blood clot DNA. DNA from (◊) vitamin B12-replete (plasma vitamin B12>148 pmol/L) and (♦) vitamin B12-deficient (plasma vitamin B12<148 pmol/L) subjects. Hashmarks to the outside of each group represent mean ± SD. Spacing along the X-axis is used only to show individual data points. See Text for further detail. (PDF) [file pone.0065241.s001.pdf]

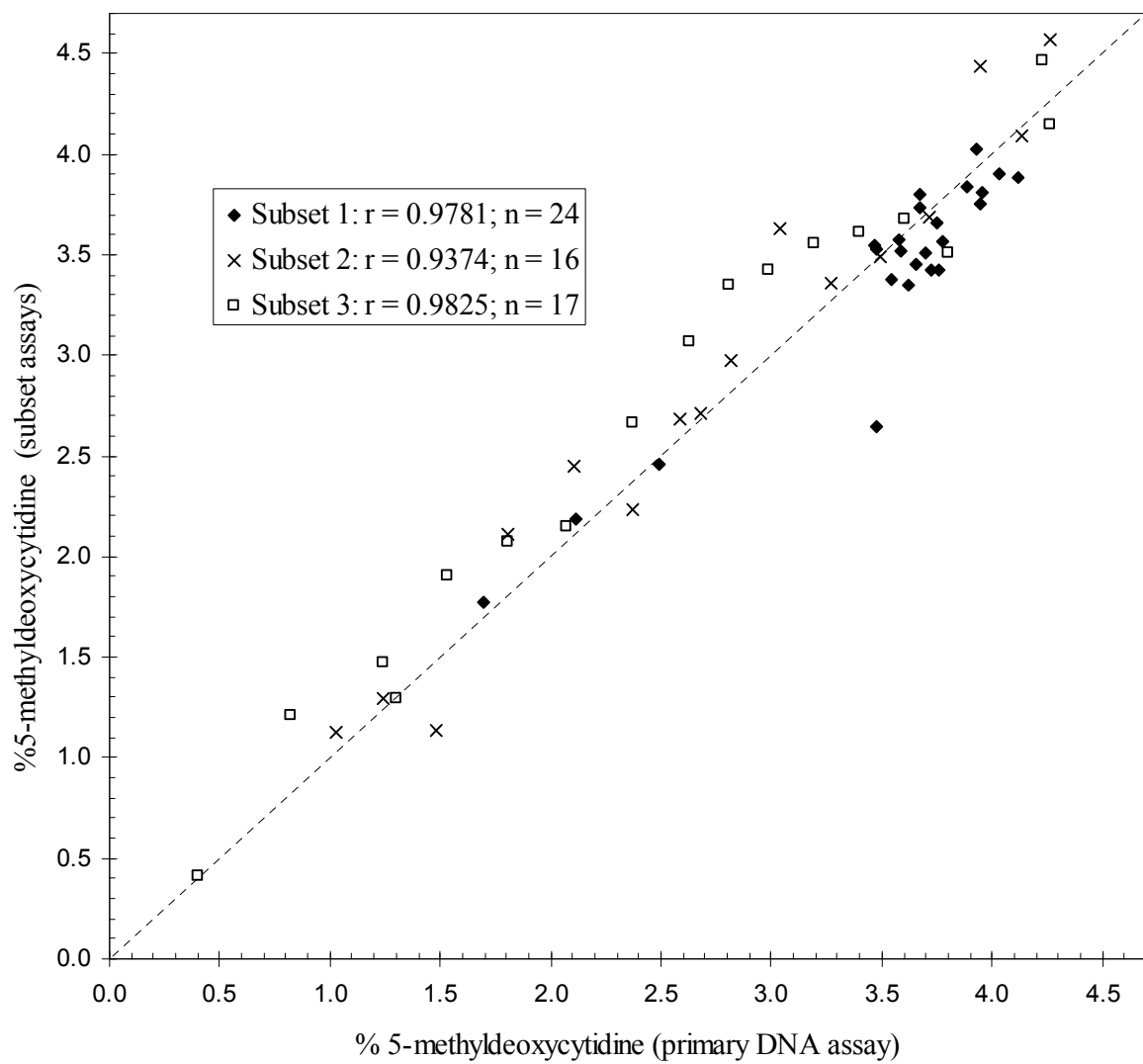

Supplement: Figure S2 — Reproducibility of the 5-methyldeoxycytidine LC-MS/MS assay. Subsets of blood clot DNA from vitamin B12-deficient subjects (plasma vitamin B12<148 pmol/L) were analyzed at different times over a two-year period and compared to results from the primary (n = 248) DNA assay. % 5-methyldeoxycytidine: 5-methyldeoxycytidine as a percentage of total deoxycytidine in DNA digests as measured by LC-MS/MS (see Methods). The broken line represents unity (X = Y). See Text for further details. (PDF) [file pone.0065241.s002.pdf]
